# Influence of age on stem cells depends on the sex of the bone marrow donor

**DOI:** 10.1111/jcmm.17201

**Published:** 2022-01-27

**Authors:** Michael Selle, Johanna Dorothea Koch, Alina Ongsiek, Linnea Ulbrich, Weikang Ye, Zhida Jiang, Christian Krettek, Claudia Neunaber, Sandra Noack

**Affiliations:** ^1^ Trauma Department Hannover Medical School Hannover Germany

**Keywords:** age, CD146, CD274, gender, mesenchymal stem cells, sex, SSEA‐4

## Abstract

Ageing is often accompanied by an increase in bone marrow fat together with reduced bone volume and diseases of the bone such as osteoporosis. As mesenchymal stem cells (MSCs) are capable of forming bone, cartilage and fat tissue, studying these cells is of great importance to understand the underlying mechanisms behind age‐related bone diseases. However, inter‐donor variation has been found when handling MSCs. Therefore, the aim of this study was to investigate the effects of donor age and sex by comparing in vitro characteristics of human bone marrow‐derived MSCs (hBMSCs) from a large donor cohort (*n* = 175). For this, hBMSCs were analysed for CFU‐F capacity, proliferation, differentiation capacity and surface antigen expression under standardized culture conditions. The results demonstrated a significantly reduced CFU‐F number for hBMSCs of female compared to male donors. Furthermore, there was a significant decrease in the proliferation rate, adipogenic differentiation potential and cell surface expression of SSEA‐4, CD146 and CD274 of hBMSCs with an increase in donor age. Interestingly, all these findings were exclusive to hBMSCs from female donors. Further research should focus on postmenopausal‐related effects on hBMSCs, as the results imply a functional loss and immunophenotypic change of hBMSCs particularly in aged women.

## 1 INTRODUCTION

One of the current major challenges in trauma surgery is the treatment of larger bone defects and cartilage damage caused by arthrosis or traumatic incident.

In addition, various studies have already shown that progressing age correlates adversely with the repair and regeneration potential of the human body.^1^, ^2^ Furthermore, there is an increasing prevalence of musculoskeletal diseases in our ageing society.^3^ As a result, interest in the therapeutic potential of human bone marrow‐derived mesenchymal stem cells (hBMSCs) has increased enormously in recent years. In this context, tissue replacement of bone and cartilage by using hBMSCs is a promising therapy option in regenerative medicine. However, while successful application of autologous MSCs has been reported in some patients,^4^, ^5^ other studies could not find clear evidence for effective treatment with hBMCs in surgery.^6^, ^7^ These contradictory results may be explained by the functional disparity observed in MSCs from different donors, including their proliferation and differentiation capacity.^8^ Some authors documented an inverse relationship between age and CFU‐F capacity^9^, ^10^, ^11^, ^12^, ^13^, ^14^ as well as proliferation rate,^11^, ^15^, ^16^, ^17^ while others found no significant evidence for such a relationship.^18^, ^19^, ^20^, ^21^ Furthermore, the expression level of some cell surface antigens, such as CD146,^22^, ^23^ CD274,^23^ SSEA‐4^24^ and others,^23^ was reported to be associated with donor age. Similarly, a correlation between the regenerative potential and the sex of MSC donors has been investigated. Some authors have found a negative correlation for female donors regarding osteogenesis and the production of collagen type I.^25^, ^26^, ^27^, ^28^ Furthermore, a correlation between oestrogen and osteogenesis with an inverse correlation to adipogenesis has been described.^29^ Other authors could not find any differences for single‐cell cloning efficiency, cumulative population doubling or colony‐forming unit‐fibroblast (CFU‐F) Assays regarding different sex.^15^, ^21^, ^30^

One reason for these contradictory results could be the heterogeneous study design of the studies conducted so far. Most of the relevant studies used BMSCs originating from either humans, mice, rats or dogs for the experiments, but some also worked with adipose tissue‐derived MSCs. Furthermore, different isolation and purification techniques with different scoring criteria were used. A decisive step towards a comparable study situation was taken by the Mesenchymal and Tissue Stem Cell Committee of the International Society for Cellular Therapy (ISCT).^31^ Here, standardized minimal criteria were introduced to characterize and define mesenchymal stem cells: MSCs have to be plastic adherent when maintained under standard culture conditions. MSCs must express CD105, CD73 and CD90 with no expression of CD45, CD34, CD14 or CD11b, CD79a or CD19 and HLA‐DR surface molecules. In addition, MSCs must differentiate into osteoblasts, adipocytes and chondrocytes in vitro.^31^

Therefore, in this study, we aimed to investigate the effects of donor age and sex on the in vitro characteristics of hBMSCs utilizing a large donor cohort (*n* = 175). For this, hBMSCs from donors (18–93 years) were harvested from iliac crest bone marrow aspirations and analysed under standardized culture conditions for CFU‐F formation, surface antigen expression of 34 selected markers, as well as for osteogenic, adipogenic and chondrogenic differentiation capacity.

## 2 MATERIALS AND METHODS

### 2.1 Recruitment of donor collective and study design

**FIGURE 1:**
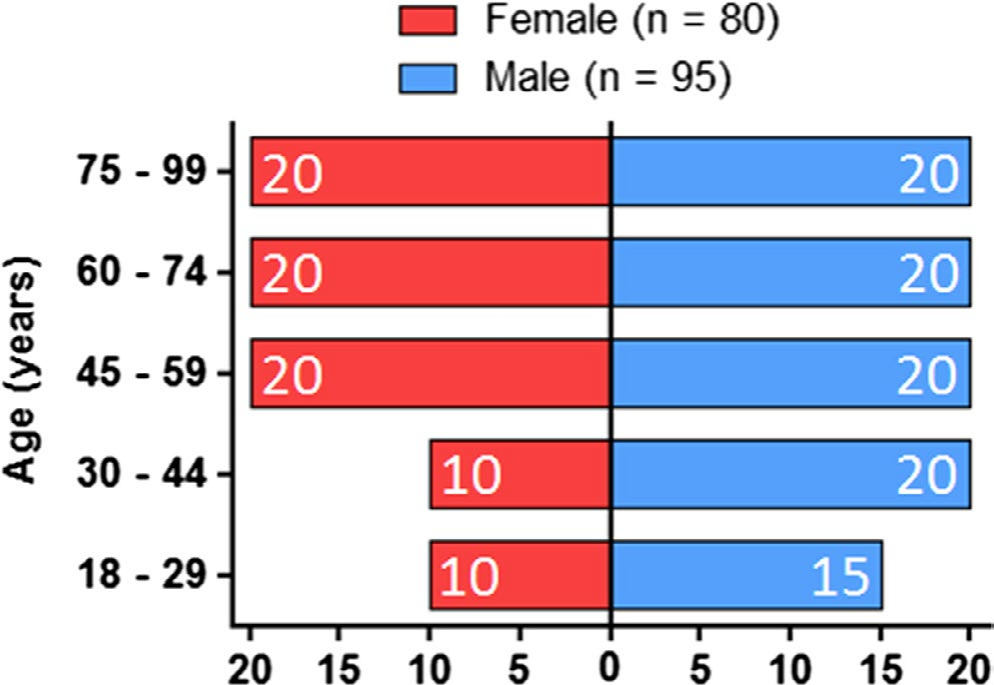
Age distribution of hBMSC donors, separately displayed for female (red; *n* = 80) and male (blue; *n* = 95) donors

The hBMSCs were obtained by bone marrow aspiration of the iliac crest from patients undergoing elective surgery at the Trauma Department of Hannover Medical School, Germany. The procedure was voluntary, and all patients gave written informed consent after being informed in detail. The study protocol and process of sample donation complied with the Declaration of Helsinki and the ethics committee of Hannover Medical School (Votum No. 2562) gave ethical approval. Inclusion criteria were age over 18 years and no infectious diseases, such as human immunodeficiency virus (HIV) or hepatitis B virus (HBV). In total, hBMSCs from 175 donors (80 female and 95 male; age ranging from 18 to 93) years were collected (Figure 1). The concomitant diseases and the medications of the bone marrow donors are summarized in Tables S1 and S2.

### 2.2 MSC isolation and cultivation

In each case, 30 mL of bone marrow was obtained from a single puncture site by a one spot aspiration technique. After acquisition, the heparinized bone marrow was mixed with phosphate buffered saline (PBS; Biochrom, Berlin, Germany) solution (1:3) and separated utilizing a synthetic polysaccharide–epichlorohydrin copolymer (Biocoll^®^, Biochrom) by centrifugation for 30 min at 500 × g without brake. Subsequently, the mononuclear cell layer was isolated, washed with PBS and centrifuged for 5 min at 500 × g with brake. The resulting cell pellet was resuspended in Dulbecco's Modified Eagle's Medium (DMEM) FG0415 (Biochrom) supplemented with 10% (v/v) Hyclone^®^ Fetal Bovine Serum (FBS; Fischer Scientific, Schwerte, Germany), 20 mM 4‐(2‐hydroxyethyl)‐1‐piperazineethanesulfonic acid (HEPES), 1% (100 U/mL/100 µg/mL) penicillin/streptomycin (P/S; Biochrom) and 2 ng/mL human recombinant fibroblast growth factor 2 (FGF‐2; PeproTech, Hamburg, Germany), hereafter called ‘complete medium’, and transferred into a culture flask. The cells were incubated in an atmosphere of 5% CO_2_ at 37°C (passage (P)0). After 24 h, the medium was changed. At the presence of at least five large adherent clones, cells were detached via Trypsin/Ethylenediamine Tetraacetic Acid (EDTA) (0.05%/0.02% (v/v)) (Biochrom) solution and reseeded at a density of 2,000 cells/cm^2^ in complete medium (P1). When a confluence of 70%–80% was reached, the cells were passaged. Next, aliquots of 1 × 10^6^ cells were transferred to 1 mL freezing medium (95% FBS and 5% dimethyl sulfoxide (DMSO; Sigma‐Aldrich)) and stored in liquid nitrogen.

### 2.3 Colony‐forming unit‐fibroblast assays and growth rate

Two CFU‐F assays were performed to investigate the self‐renewal potential of the cells. The first CFU‐F assay was performed with hBMSCs in P1, the second with hBMSCs in P3. For this, the hBMSCs were seeded at three different densities (125, 250 and 500 per well) as duplicates in 6‐well plates (Greiner Bio‐one, Frickenhausen, Germany). After an incubation period of 10 days at 37°C and 5% CO_2,_ cells were fixed with 100% methanol (J.T. Baker, Pennsylvania, USA) for 30 min, washed with water and stained with 1% (w/v) crystal violet (Merck, Darmstadt, Germany) for 30 min. Afterwards, macroscopically visible colonies were counted, and the average number of colonies per 100 cells seeded (hereafter termed as ‘% CFU‐F’) was calculated. In the literature, CFU‐F is defined as a number of 50 or more clonal cells.^32^

To estimate the proliferation of hBMSCs in P1, the growth rate was calculated as demonstrated in the following formula:

$${\mathrm{Doubling}\;\mathrm{Time}\;(T}_{d})=\frac{\ln\left(2\right)∙d}{\ln(\frac{N_{d}}{N_{0}})}$$

with N_0_ = cells seeded at P1, N_d_ = cells counted at the end of P1, d = days in culture during P1.

### 2.4 Differentiation

Adipogenic, chondrogenic and osteogenic differentiation assays were performed in P4. Therefore, cryo‐conserved hBMSCs were thawed and expanded in complete medium. Then, 150,000 cells per 9.6 cm² were seeded into 6‐well plates. After 24 h, the complete medium was replaced with the according differentiation or control media (day 0). For osteogenic differentiation, DMEM FG0415 containing 0.1 µM dexamethasone (Sigma‐Aldrich, Taufkirchen, Germany), 50 µM ascorbate‐2‐phosphat (Sigma‐Aldrich), 3 mM disodium hydrogen phosphate (Merck), 20 mM HEPES, 10% FBS and 1% P/S was used. For adipogenic differentiation, DMEM FG0435 (Biochrom) containing 1 µM dexamethasone, 60 µM indomethacin (Sigma‐Aldrich), 500 µM 3‐isobutyl‐1‐methylxanthin (Sigma‐Aldrich), 10 µg/mL insulin (Sigma‐Aldrich), 20 mM HEPES, 20% FBS and 1% P/S was used. The control medium for osteogenesis and adipogenesis consisted of DMEM FG0415 supplemented with 20 mM HEPES, 10% FBS and 1% P/S.

For the chondrogenic differentiation, a cell pellet was formed by centrifugation of 250,000 cells for 5 min at 500 × g and then cultivated in conical tubes at 37°C and 5% CO_2_. The complete medium was replaced by differentiation or control media after 24 h (day 0). The differentiation medium consisted of DMEM FG0435 including 20 mM HEPES, 1% P/S, 0.1 μM dexamethasone, 10 μL/mL insulin/transferrin/selenium (Sigma‐Aldrich), 170 µM ascorbate‐2‐phosphate, 1 mM sodium pyruvate (Biochrom), 350 μM proline (Carl Roth, Karlsruhe, Germany) and 10 ng/mL transforming growth factor beta‐3 (TGF‐β3; PeproTech). The control medium did not contain TGF‐β3. The media for all the groups were replaced every 7 days, and the differentiations were stopped after 28 days.

### 2.5 Histological staining and interpretation

For histological staining, cells were fixed with 4% formalin (Medite, Burgdorf, Germany). Calcium ions from osteogenic differentiation were stained with Alizarin Red (Roth, 0.5% (w/v) in water) for 10 min, while lipids from adipogenic differentiation were stained with Oil Red O (Sigma‐Aldrich, 5 g/L in 60% (w/v) isopropanol) for 25 min.

The cell pellets from chondrogenic differentiation were embedded in Tissue‐Tek (Sakura Finetek, Staufen, Germany) frozen in liquid nitrogen and stored at −20°C until use. The pellets were cut into 5 µm thin slices using a cryotome (CM 3050S, Leica Biosystems, Wetzlar, Germany) and transferred to adhesively coated slides (SuperFrost Plus, Fisher Scientific). Next, proteoglycans contained within the cartilage matrix were stained for 15 min with Safranin O (Merck, 0.1% (w/v) in water), after which the slices were embedded in Vitro‐Clud (R. Langenbrinck, Emmendingen, Germany).

Finally, representative images were taken using light microscopes considering osteogenic, adipogenic (CKX41, Olympus, Hamburg, Germany) and chondrogenic (BX41, Olympus) differentiation. The degree of osteogenic and adipogenic differentiation was determined by calculating the average of the stained area relative to the total area. To obtain a valid result, this procedure was applied to three representative images of the respective populations. To facilitate this task, a self‐written tool for image processing, relying on the OpenCV library (version 4.1.0), was used. With the help of the software tool, the stained area was distinguished from the background area based on different ‘hue’ and ‘saturation’ values. The thresholds for these parameters were set manually using specific characteristics from a representative image and then applied automatically to all images for uniform scoring.

Three members of the laboratory (M.S., W.Y and Z.J.) assessed the average amount of chondrogenesis manually for each sample independently. Within one image, the areas showing chondrogenic cell differentiation were manually defined and quantified in relation to the total area of the pellets. All analyses were performed blindly without prior knowledge to underlying donor data.

### 2.6 Flow cytometry

The hBMSCs were analysed in P4 for the surface expression of 34 antigens via flow cytometry. These included antibodies for CD73, CD90 and CD105 as positive markers as well as CD11b, CD14, CD19, CD34, CD45 and HLA‐DR as negative markers in accordance with the minimal criteria for MSCs as suggested by the ISCT.^31^ Apart from these, the following antigens were also analysed in order to search for age‐ and sex‐specific differences between the hBMSC populations: CD4, CD10, CD11c, CD13, CD15, CD29, CD31, CD44, CD49f, CD56, CD106, CD117, CD146, CD163, CD166, CD200, CD271, CD274, GD2, MSCA‐1, SSEA‐3, SSEA‐4, SSEA‐5, CD24 and Stro‐1. More detailed information about each antibody is listed in Table S3.

For flow cytometry, cells were detached using 0.025% trypsin‐EDTA solution and washed twice with FC buffer [2% (v/v) FBS in PBS]. All centrifugation steps were performed at 4°C at 400 × g for 2 min. For each approach, 1 × 10^5^ cells were used and incubated with appropriate fluorochrome‐conjugated antibodies as shown in Table S3 for 60 min at 4°C in the dark. Afterwards, cells were washed twice with FC buffer. For analysis, a FACS Canto (BD Biosciences, Heidelberg, Germany) was used as described before by Schäck et al.^8^ Briefly, each analysis consisted of a record of 3 × 10^4^ cells. Dead cells were excluded by using scatter parameters in BD FACS Diva Software and Flowing Software version 2.5.0.

### 2.7 Statistics

The statistical analyses were performed with IBM SPSS Statistics software (version 26). The data were first tested for distribution using the Shapiro–Wilk test and found to be non‐normally distributed (*p* < 0.05). The Mann–Whitney *U*‐test was used to compare data from female and male donors. Correlation analyses were done with the two‐tailed Spearman test. The figures were created with GraphPad Prism software (version 5). The box plots represent median values with whiskers plotted after the Tukey method. Data are described as median (Mdn) together with interquartile range (IQR). A *p*‐value < 0.05 was considered statistically significant.

## 3 RESULTS

### 3.1 Colony‐forming unit‐fibroblast assay and population doubling time

**FIGURE 2:**
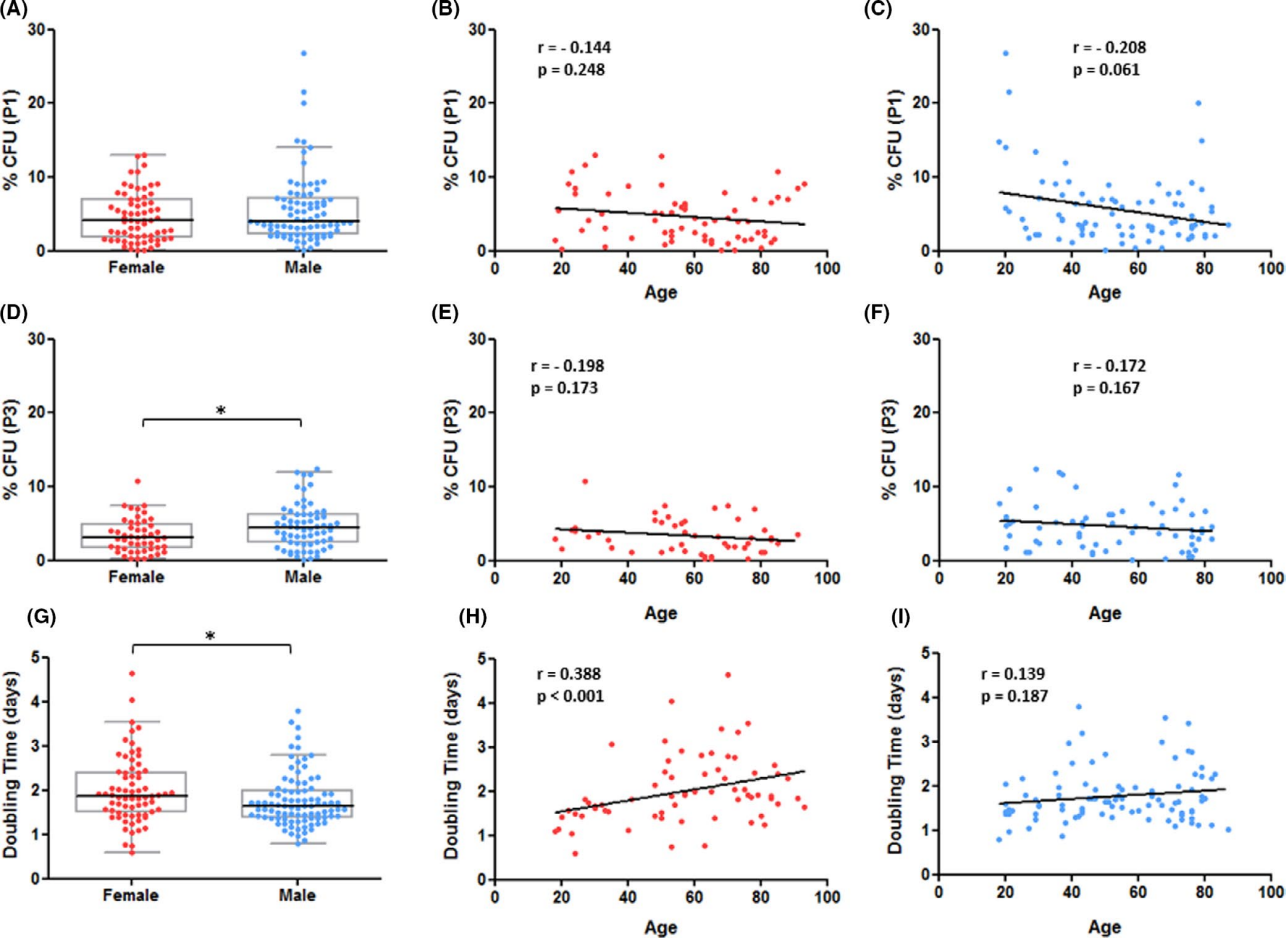
Average number of CFU‐F per 100 cells seeded (% CFU‐F) and doubling time in relation to donor gender and age. (A, D): Comparison of % CFU‐F in P1 and P3 between genders, independent from donor age. The CFU‐F number in P3 was significantly reduced in hBMSCs from female donors. (B, C, E and F): Analyses of correlation between % CFU‐F and donor age, divided by gender. There was no correlation between % CFU‐F and age, neither in female or male donors nor in P1 and P3. Data displayed separately for hBMSCs from female (red; *n* = 66 in P1, *n* = 49 in P3) and male (blue; *n* = 82 in P1, *n* = 66 in P3) donors. (G): Comparison of doubling time in P1 between genders, independent from donor age. The doubling time was significantly higher in hBMSCs from female donors. (H, I): Analyses of correlation between doubling time and donor age, divided by gender. In hBMSCs from female donors, the doubling time was significantly correlated with donor age, whereas there was no significant correlation for male donors. Data displayed separately for hBMSCs from female (red; *n* = 70) and male (blue; *n* = 92) donors. Statistically significant differences are shown as *p < 0.05

A CFU‐F assay was performed in P1 and P3. Notably, the colonies varied a lot in size and density (data not shown). In P1, there was no significant difference in % CFU‐F number between female and male donors (Mdn = 4.14%, IQR = 5.24% vs. Mdn = 4.05%, IQR = 4.84%, *p* = 0.238; Figure 2A). On the contrary, the hBMSCs from female donors formed significantly less CFU‐F in P3 in contrast to male donors (Mdn = 3.10%, IQR = 3.30% vs. Mdn = 4.40%, IQR = 3.93%, *p* = 0.024; Figure 2D). On average, for hBMSCs from female and male donors the % CFU‐F number was 1.38‐fold and 1.22‐fold higher in P1, as compared to P3 respectively. No age‐related changes were found for % CFU, independent of sex (Female: Figure 2B and E; Male: Figure 2C and F) or passage (P1: Figure 2B and C; P3: Figure 2E and F).

The population doubling time of hBMSCs was estimated during culture in P1. A significantly higher doubling time was found in hBMSCs from female donors than in hBMSCs from male donors (Mdn = 1.88 days, IQR = 0.91 days vs. Mdn = 1.63 days, IQR = 0.61 days, *p* = 0.017; Figure 2G). There was a significant positive correlation between doubling time and donor age in hBMSCs from female donors (*r* = 0.388, *p* < 0.001; Figure 2H), but not in hBMSCs from male donors (*r* = 0.139, *p* = 0.187; Figure 2I).

### 3.2 Differentiation potential

**FIGURE 3:**
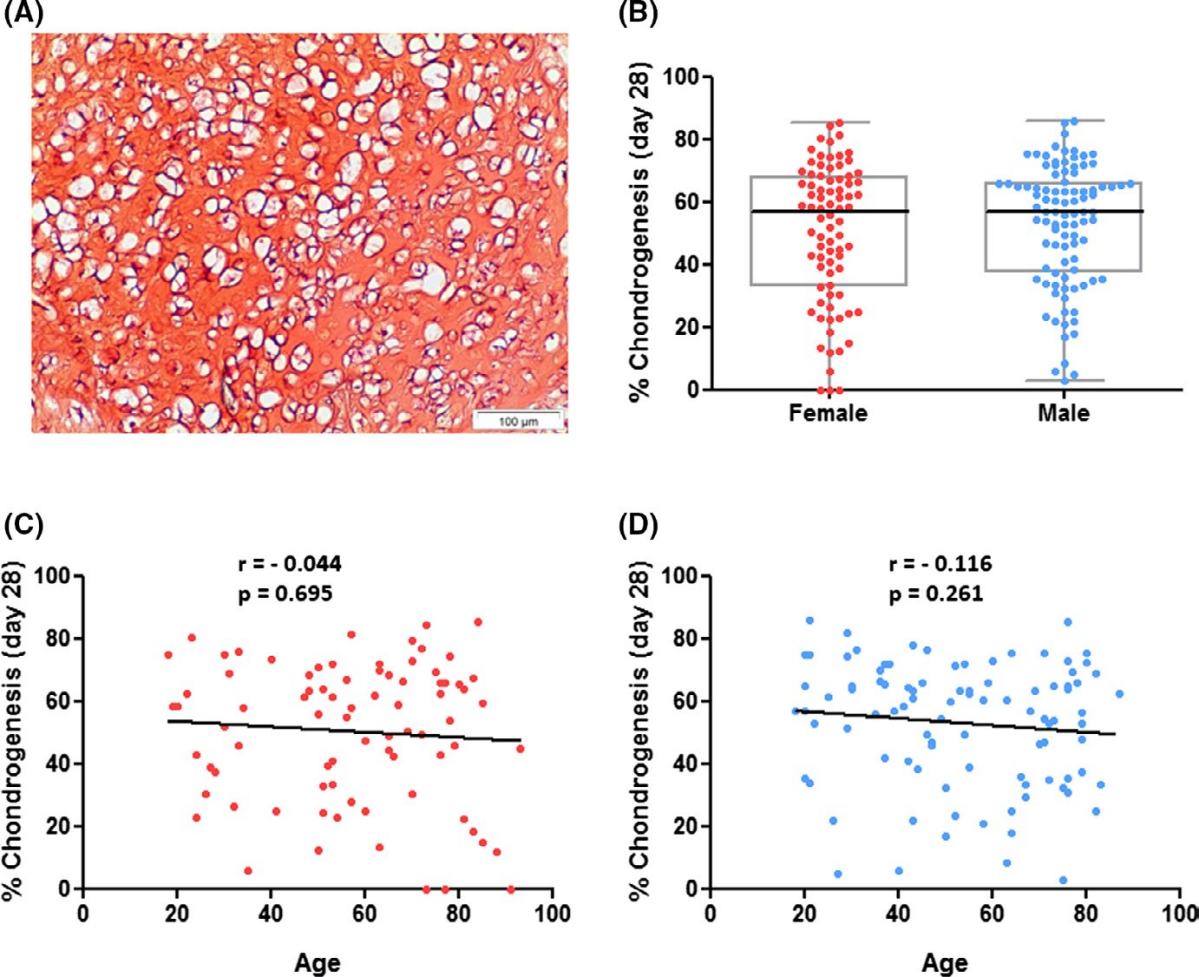
In vitro chondrogenesis of hBMSCs in P4 in relation to donor gender and age. (A): Representative image of induced chondrogenesis in hBMSCs after 28 days of culture in chondrogenic differentiation medium showing chondrocytes with an extracellular proteoglycan matrix stained by Safranin O. Scale bar = 100 µm. (B): Comparison of chondrogenesis between genders, independent from donor age. (C, D): Analyses of correlation between chondrogenesis and donor age, divided by gender. No gender‐ and age‐dependent relationships were found. Data displayed separately for hBMSCs from female (red; *n* = 80) and male (blue; *n* = 95) donors

**FIGURE 4:**
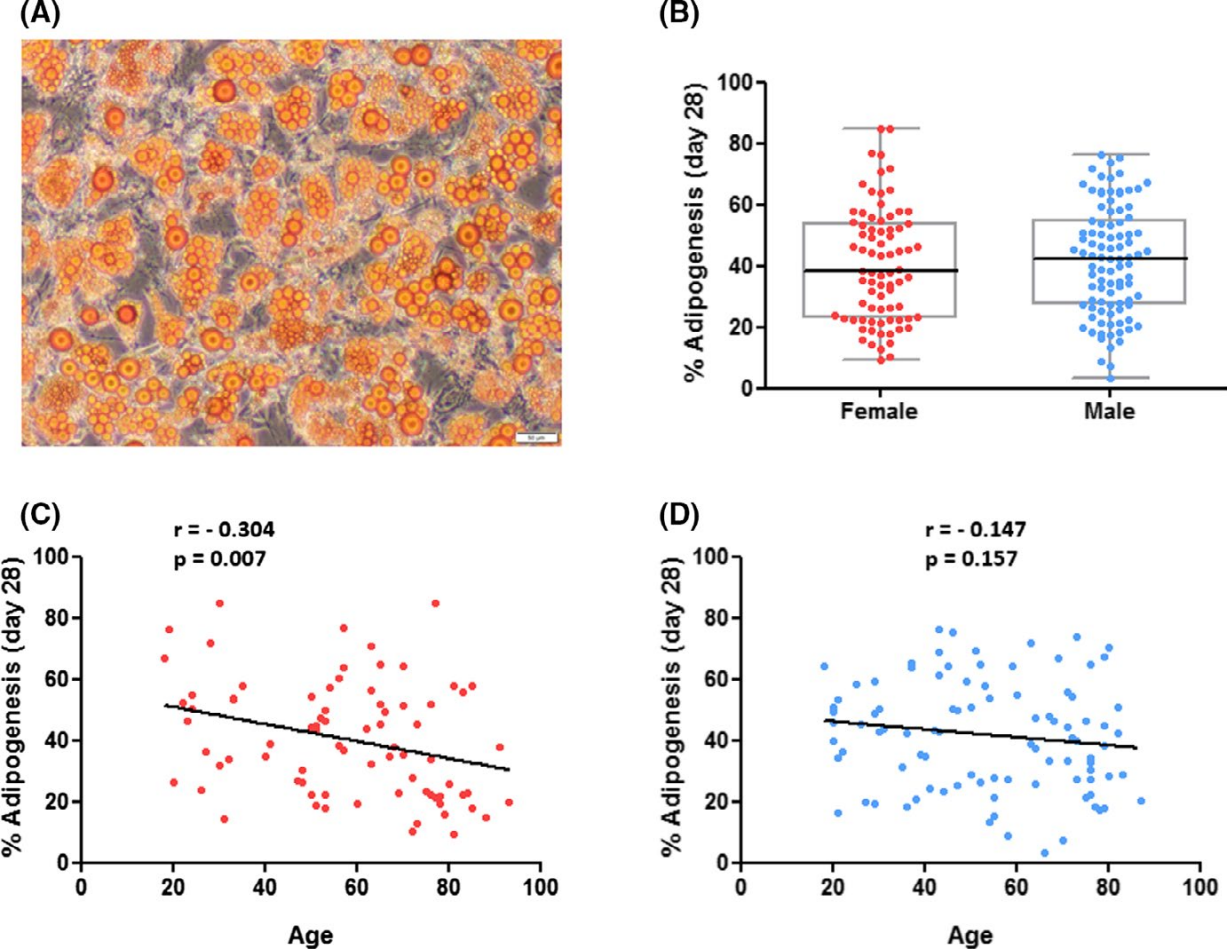
In vitro adipogenesis of hBMSCs in P4 in relation to donor gender and age. (A): Representative image of induced adipogenesis in hBMSCs after 28 days of culture in adipogenic differentiation medium showing intracellular oil droplets from adipocytes stained by lipophilic Oil Red O. Scale bar = 50 µm. (B): Comparison of adipogenesis between genders, independent from donor age, with no differences between hBMSCs from female and male donors. (C, D): Analyses of correlation between adipogenesis and donor age, divided by gender, demonstrating a significant negative correlation for hBMSCs from female donors. Data displayed separately for hBMSCs from female (red; *n* = 78) and male (blue; *n* = 94) donors

The hBMSCs were able to differentiate in vitro according to three lineages (Figure 3A, 4A, 5A) to varying degrees.

**FIGURE 5:**
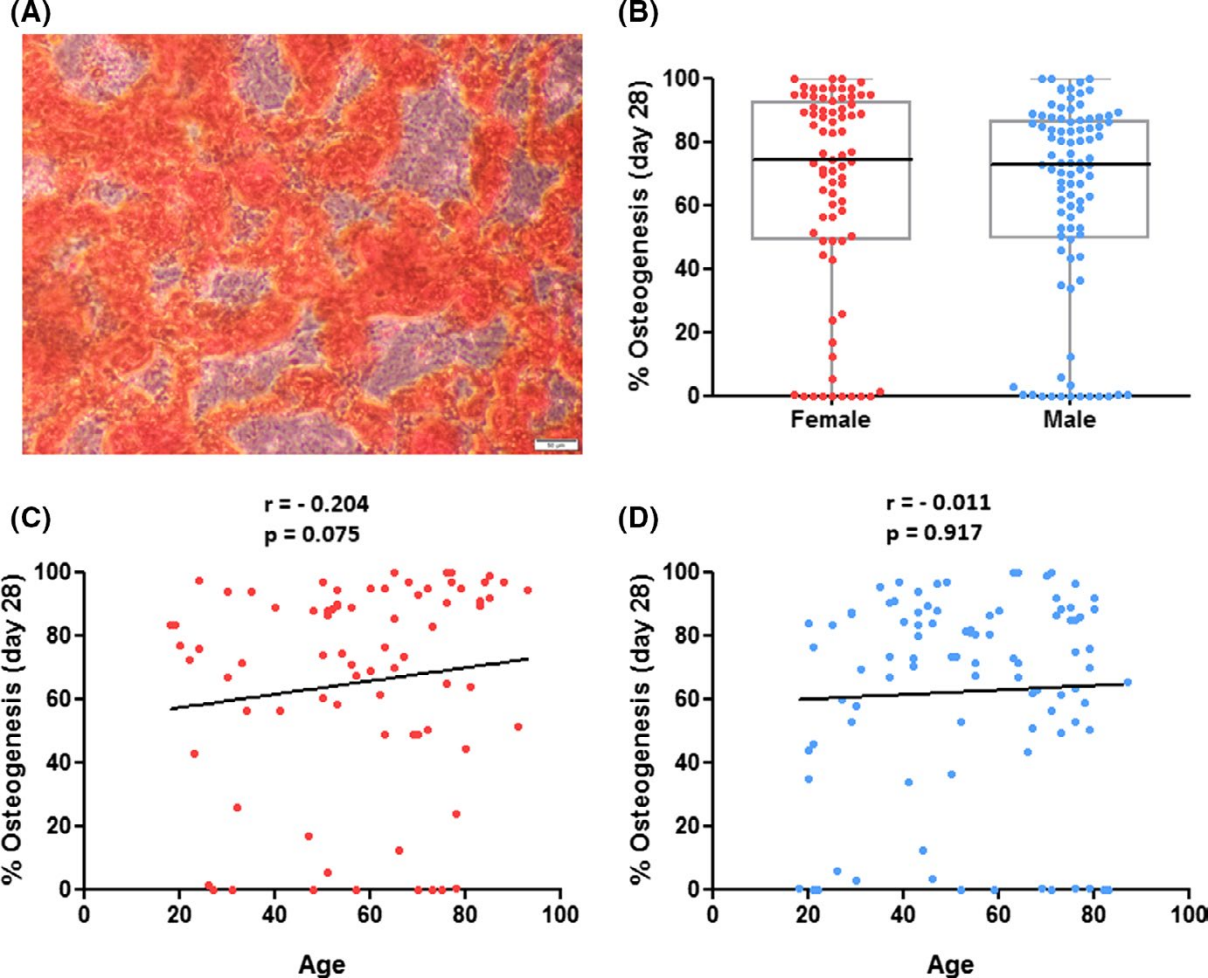
In vitro osteogenesis of hBMSCs in P4 in relation to donor gender and age. (A): Representative image of induced osteogenesis in hBMSCs after 28 days of culture in osteogenic differentiation medium showing mineralized osteoblasts with extracellular calcium deposits stained by Alizarin Red. Scale bar = 50 µm. (B): Comparison of osteogenesis between genders, independent from donor age. (C, D): Analyses of correlation between osteogenesis and donor age, divided by gender. No gender‐ and age‐dependent relationships were found. Data displayed separately for hBMSCs from female (red; *n* = 77) and male (blue; *n* = 92) donors

The median amount of chondrogenesis was 56.83% (IQR = 34.82%) in hBMSCs from female donors and 56.82% (IQR = 28.46%) in hBMSCs from male donors (Figure 3B). Therefore, the in vitro chondrogenic differentiation capacity was neither influenced by sex (Figure 3B) nor correlated to donor age (Figure 3C,D).

The capacity of the cells to differentiate into adipocytes in vitro was comparable between hBMSCs from female and male donors (Mdn = 38.22%, IQR = 30.97% vs. Mdn = 42.51%, IQR = 27.87%, *p* = 0.528; Figure 4B). However, in hBMSCs from female donors the amount of adipogenesis significantly decreased with an increase in donor age (*r* = −0.304, *p* = 0.007; Figure 4C), whereas in hBMSCs from male donors no significant alterations with age were found (*r* = −0.147, *p* = 0.157; Figure 4D). Sometimes, mild spontaneous adipogenesis occurred inside the control group, unrelated to donor age or sex: hBMSCs from 27 female donors (33.75%) and 25 male donors (26.32%) formed lipid droplets in the absence of differentiation medium (data not shown).

No significant difference was found in regard to the quantity of in vitro osteogenesis of hBMSCs between female and male donors on day 28 (Mdn = 74.44%, IQR = 43.29% vs. Mdn = 72.88%, IQR = 37.12%, *p* = 0.296; Figure 5B). Furthermore, there was no significant correlation of osteogenesis and age, neither in hBMSCs from female (Figure 5C) nor from male donors (Figure 5D).

### 3.3 Surface antigen markers

The hBMSCs were screened for the surface expression of 34 distinct antigens in P4 via flow cytometry in search for sex‐ and age‐associated differences in their surface expression profiles (Tables 1, S4). CD13, CD29, CD44, CD73, CD90, CD105, CD166 and GD2 were highly expressed (Mdn > 90%) irrespective of age or sex. There was no too little expression (Mdn < 10%) of CD4, CD11b, CD11c, CD14, CD15, CD19, CD31, CD45, CD54, CD117, CD163, CD271, MSCA‐1, SSEA‐3, SSEA‐5 and Stro‐1. With regard to the results of flow cytometry, some of the surface antigens examined showed a wide range of variation with regard to the expression rate. These were classified as variable markers. In this study variable, expression on the cell surface of hBMSCs was found for CD10, CD34, CD49f, CD56, CD106, CD146, CD200, CD274, HLA‐DR and SSEA‐4.

**TABLE 1:** Cell surface expression of 34 selected markers for hBMSCs from female and male donors

	Surface antigen	Female	Male	Surface antigen	Female	Male	Surface antigen	Female	Male
*n*	CD4	69	90	CD45	80	95	CD200	80	95
Median (IQR)		8.45 (6.12)	7.56 (5.20)		1.99 (2.38)	2.00 (1.95)		17.47 (22.22)	21.73 (23.62)
*n*	CD10	80	95	CD49f	80	95	CD271	80	95
Median (IQR)		13.03 (11.21)	14.39 (13.83)		30.91 (28.47)	40.40 (27.78)		2.88 (3.69)	2.76 (2.90)
*n*	CD11b	80	95	CD54	70	90	CD274	72	89
Median (IQR)		1.20 (0.59)	1.30 (0.57)		3.52 (3.44)	2.44 (3.02)		11.27 (26.37)	14.28 (31.02)
*n*	CD11c	80	95	CD56	80	95	GD2	80	95
Median (IQR)		2.88 (3.61)	2.51 (2.61)		40.48 (20.39)	47.50 (22.22)		96.07 (5.55)	94.49 (6.50)
*n*	CD13	80	95	CD73	80	95	HLA‐DR	74	88
Median (IQR)		99.06 (8.09)	98.18 (15.91)		98.18 (3.98)	97.89 (5.12)		73.18 (33.39)	87.06 (29.67)
n	CD14	80	95	CD90	80	95	MSCA‐1	80	95
Median (IQR)		1.52 (1.80)	1.66 (0.95)		97.57 (4.36)	96.38 (4.92)		9.33 (11.48)	8.24 (7.83)
*n*	CD15	77	91	CD105	80	95	SSEA‐3	75	88
Median (IQR)		0.98 (0.38)	0.95 (0.28)		99.52 (0.82)	99.47 (0.63)		1.20 (0.70)	1.20 (0.42)
*n*	CD19	71	90	CD106	80	95	SSEA‐4	73	86
Median (IQR)		2.70 (1.62)	2.67 (1.52)		54.11 (37.02)	54.01 (37.62)		72.46 (32.41)	79.80 (22.93)
*n*	CD29	80	95	CD117	80	95	SSEA‐5	80	95
Median (IQR)		98.27 (3.63)	98.95 (2.85)		1.45 (1.07)	1.47 (0.87)		3.53 (4.39)	4.27 (7.12)
*n*	CD31	80	95	CD146	80	95	Stro‐1	80	95
Median (IQR)		1.29 (0.69)	1.28 (0.57)		61.81 (30.09)	63.00 (22.05)		1.03 (0.44)	1.06 (0.47)
*n*	CD34	71	90	CD163	75	88			
Median (IQR)		12.83 (10.90)	13.51 (10.77)		1.29 (1.01)	1.28 (0.72)			
*n*	CD44	80	95	CD166	80	95			
Median (IQR)		94.91 (8.06)	96.30 (8.44)		97.86 (4.17)	98.15 (2.85)			

**FIGURE 6:**
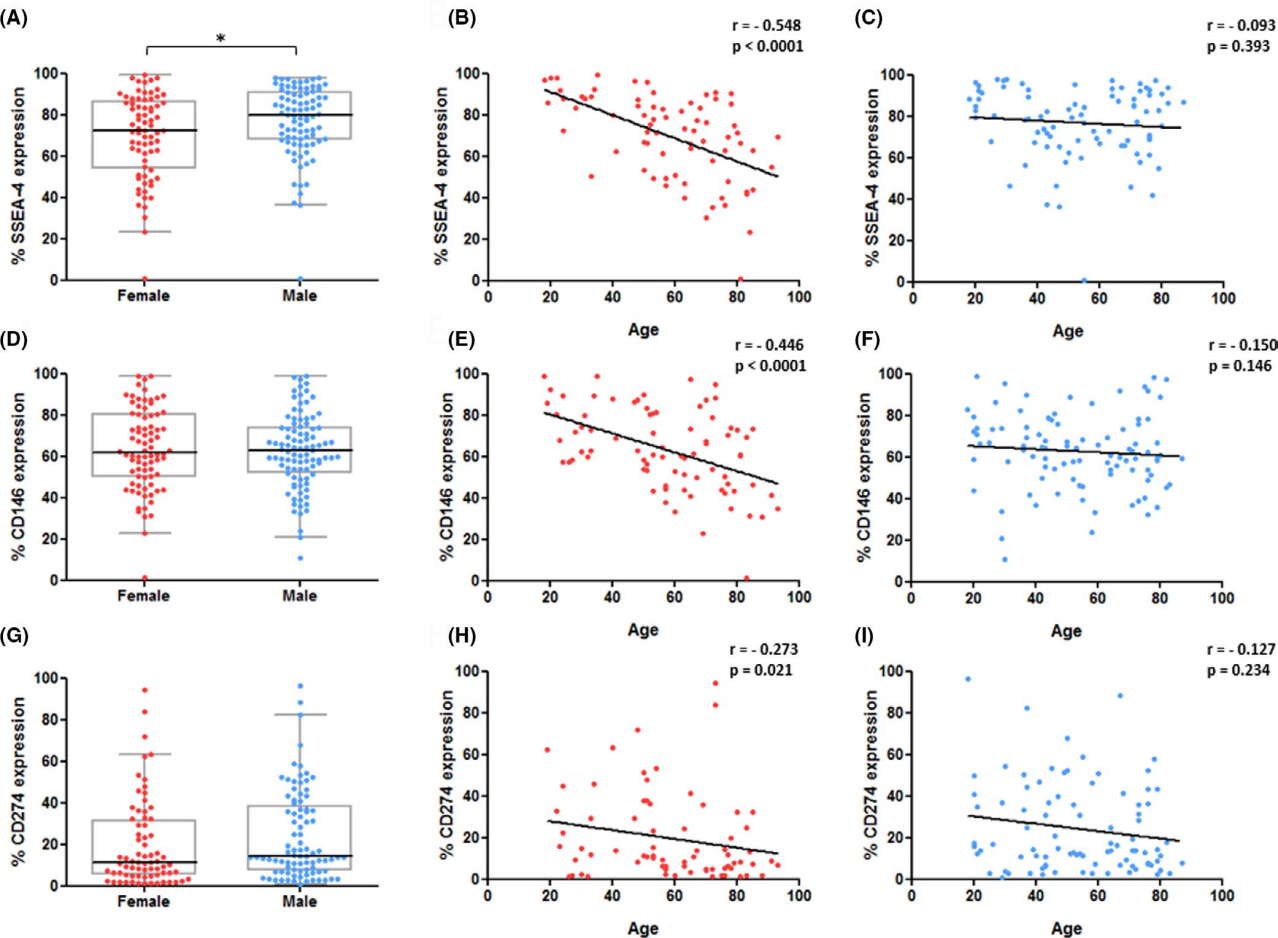
Expression levels of SSEA‐4, CD146 and CD274 on the cell surface of hBMSCs in relation to donor gender and age. (A–C): Expression of SSEA‐4 was significantly reduced on hBMSCs from female donors and significantly declined with an increase in donor age for female donors. (D–F): CD146 significantly declined with an increase in donor age on hBMSCs from female donors. (G–I): CD274 significantly declined with an increase in donor age on hBMSCs from female donors. Data displayed separately for hBMSCs from female (red; *n* = 73, 80, 72 for SSEA‐4, CD146 and CD274 respectively) and male (blue; *n* = 86, 95, 89 for SSEA‐4, CD146 and CD274 respectively) donors. Statistically significant differences are shown as *p < 0.05

Correlation analyses revealed that some of these surface antigens were found to be expressed in an age‐dependent manner, namely SSEA‐4, CD146 and CD274. SSEA‐4 expression on the cell surface of hBMSCs was significantly lower in female donors in comparison with male donors (Mdn = 72.46%, IQR = 32.41% vs. Mdn = 79.80%, IQR = 22.93%, *p* = 0.034; Figure 6A). Furthermore, the expression of SSEA‐4 on hBMSCs from female donors declined significantly with increasing donor age (*r* = −0.548, *p* < 0.0001; Figure 6B). In contrast, no significant correlation between SSEA‐4 expression and donor age was found for hBMSCs from male donors (*r* = −0.093, *p* = 0.393; Figure 6C). There was no difference in the expression level of CD146 when comparing female and male donors (Mdn = 61.81%, IQR = 30.09% vs. Mdn = 63.00%, IQR = 22.05%, *p* = 0.803; Figure 6D). Nonetheless, CD146 expression on the cell surface of hBMSCs from female donors significantly diminished with increasing donor age (*r* = −0.446, *p* < 0.0001; Figure 6E), whereas in male donors, the expression of CD146 was unaffected by donor age (*r* = −0.150, *p* = 0.146; Figure 6F). CD274 expression levels did not significantly differ between female and male donors (Mdn = 12.63%, IQR = 32.10% vs. Mdn = 16.09%, IQR = 35.41%, *p* = 0.186; Figure 6G). Yet, on hBMSCs from female donors the expression of CD274 negatively correlated with donor age (*r* = −0.273, *p* = 0.021; Figure 6H), while once again on hBMSCs from male donors no significant correlation was found (*r* = −0.127, *p* = 0.234; Figure 6I).

Beyond that, further statistical significances were uncovered, although with differences in expression levels smaller than 5%, which are not listed in this manuscript as they are of no clinical relevance for us (e.g. the expression of GD2 was significantly higher on the cell surface of hBMSCs from female donors compared to male donors with Mdn = 96.07%, IQR = 5.55% vs. Mdn = 94.49%, IQR = 6.50%, *p* = 0.028 respectively).

## 4 DISCUSSION

The aim of this study was to assess inter‐donor variability of hBMSCs based on donor age and sex.

The decline in CFU‐F potential of hBMSCs over passages in culture as observed in the present study by comparing CFU‐F forming capacity between P1 and P3 is in agreement with studies by other authors and has been associated with accelerated culture‐induced in vitro ageing of MSCs, for example by shortening of telomeres and a higher proportion of senescent cells with continued population doublings, as reviewed by Ganguly et al.^1^ The present study shows no correlation between donor age and CFU‐F capacity, fitting the results obtained by few others.^18^, ^33^ In turn, most studies reported a decrease in CFU‐F frequency with increasing donor age^11^, ^23^, ^24^, ^34^, ^35^, ^36^ (additional findings up to 2006 summarized by Sethe et al.^2^). These contradictory results may presumably be explained by the lack of standardization of the CFU‐F protocols.^2^ While a large number of studies have analysed the correlation between age and CFU‐F potential, sex‐related effects have been sparsely studied and have also shown conflicting results. Some studies reported no differences between sexes^15^, ^34^; others, however, such as Katsara et al., found significantly less CFU‐F in murine BMSCs from female donors of all age groups after seeding of freshly isolated bone marrow mononuclear cells.^36^ Siegel et al. noted a significantly higher CFU‐F frequency in hBMSCs from female donors in P1.^23^ Our own results show no sex‐dependent differences for CFU‐F in P1 but a significantly decreased CFU‐F frequency in hBMSCs from female donors in P3, implying faster in vitro ageing of hBMSCs from female donors.

Most studies demonstrated a decline or no change in the proliferation rate of BMSCs from young donors to old donors (as reviewed by Baker et al.^37^), as well as no differences between female and male donors.^15^, ^30^ Our data show a significant decline in the proliferation rate, here displayed as an increase in population doubling time, of hBMSCs in P1 with increasing donor age. Interestingly, when analysing the population doubling time separately for female and male donors, an age‐dependent increase was solely found in hBMSCs from female donors. Regarding sex‐dependent effects, Siegel et al. found—opposing to our own results—a lower population doubling time in hBMSCs from female donors compared to male donors, albeit comparing much smaller sample sizes. Furthermore, in conformance with our own results, they also found an inverse relationship between the population doubling time and number of CD146^+^ cells.^23^

Age‐dependent reduction in bone mass and deterioration of the bone microarchitecture are hallmarks of senile osteoporosis, and are associated with an imbalance in bone remodelling. Since there is an abundance of fat tissue in the adult bone marrow and BMSCs give rise to both adipocytes and osteoblasts, it is expected that among other factors age‐related diseases of the bone are caused by a shift of BMSC differentiation towards the adipogenic lineage and an increase in senescent cells.^38^, ^39^, ^40^ Therefore, it seems plausible that BMSCs from the elderly tend towards adipogenic differentiation with impairment in osteogenic differentiation or even in their overall differentiation capacity. Surprisingly, in the present study, both the chondrogenic and the osteogenic potential remained unchanged, irrespective of age and sex. On the contrary, the adipogenic potential significantly decreased with an increase in donor age in hBMSCs from female donors, showing once again a sex bias in respect to the influence of age on hBMSCs. Several studies reported no changes in the in vitro adipogenic,^15^, ^23^, ^34^, ^41^, ^42^ osteogenic^15^, ^23^, ^34^, ^41^, ^42^, ^43^ or chondrogenic^15^, ^19^, ^23^, ^29^, ^34^, ^42^ differentiation capacity of BMSCs from different species regardless of age or sex. Sethe et al. listed a variety of older literature mostly reporting an age‐related decrease in either adipogenesis or osteogenesis while few publications reported an age‐related increase in adipogenesis or no age‐related changes.^2^ Results in respect to sex‐specific influence of the donor age on hBMSC properties were reported by Payne et al., who found chondrogenesis, assessed by pellet size, histological grading and glycosaminoglycan content, to decrease specifically in human femoral bone‐derived MSCs from male but not from female donors with an increase in donor age.^30^ Also, further aspects, such as the ageing of the whole body and thus changes in the microenvironment surrounding hBMSCs, have to be taken into account. For example, Singh et al. have shown that the in vivo microenvironment of older mice favours differentiation of mBMSCs towards adipogenesis independent from donor age,^44^ while Ganguly et al. have shown that serum from younger donors could positively affect the in vitro proliferation of hBMSCs from older donors.^34^ This, in turn, might signify that donor age and sex are of less significance when using hBMSCs as therapeutic agents in regenerative strategies.

In our study, SSEA‐4, CD146 and CD274 expression on hBMSCs decreased with an increase in donor age. This is mainly in line with findings by other authors, as SSEA‐4, CD146 and CD274 have already been acknowledged as markers of MSC ageing in the literature.^22^, ^23^, ^24^, ^45^ Interestingly, the present study is the first to suggest that this effect may be more pronounced in hBMSCs from female donors. SSEA‐4 was first proposed as a potential marker for a potent subpopulation of MSCs by Gang et al. in 2007.^46^ A decrease in SSEA‐4 expression with increasing donor age has recently been described for hBMSCs^24^ and human periodontal‐ligament‐derived stem cells.^47^ Block et al. managed to isolate a subpopulation of small‐sized SSEA‐4^+^ hBMSCs from elderly male patients with a ‘youthful’ phenotype. These resembled hBMSCs from younger patients, which displayed superior proliferation and differentiation potential as well as less pronounced signs of cell senescence.^24^ Rosu‐Myles et al. described a decrease of SSEA‐4 expression during in vitro expansion but further informed that the level of SSEA‐4 expression also varies between donors. Furthermore, they reported that the expression of SSEA‐4 was higher using culture media without serum.^48^ CD146, also known as melanoma‐associated cell adhesion molecule (MCAM), is regarded as a candidate antigen for describing potent MSC subpopulations.^49^, ^50^, ^51^ In line with our results, a negative correlation between donor age and CD146 expression on hBMSCs was presented in earlier studies,^22^, ^23^ although without investigating sex‐dependency. Ganguly et al. found no differences in CD146 expression on the surface of CD271^+^/CD45^−^ sorted hBMSCs from young and old donors, omitting a comparison between female and male donors.^34^ Interestingly, it was shown that CD146 expression on hBMSCs decreases during in vitro expansion in DMEM.^52^ CD274, also termed Programmed Cell Death 1 Ligand 1 (PDL1), is a type I transmembrane protein which functions as an inhibitory regulator of immune responses.^53^ An inverse relationship of donor age with CD274 expression on hBMSCs has been described before,^23^ which is in agreement with the results presented here. Interestingly, our research group has found a significantly reduced expression of CD274 on hBMSCs from osteoporosis patients in comparison with hBMSCs from sex‐ and age‐matched control donors (*n* = 7 per group).^54^

Except for the negative markers CD34 and HLA‐DR, the expression of the investigated positive (CD73, CD90 and CD105) and negative markers (CD11b, CD14, CD19 and CD45) were in compliance with the minimal criteria for MSCs proposed by the ISCT.^31^ CD34 was found to be expressed at low levels on the cell surface of hBMSCs from some donors. Lin et al. questioned the idea of CD34 being a negative marker, as several studies isolated hBMSCs based on CD34^+^ expression and its negative status has been reported to be due to cell culture associated loss of CD34 expression on hBMSCs.^55^ HLA‐DR was generally expressed on the cell surface of hBMSCs from all donors in this study. Bocelli‐Tyndall et al. have shown that HLA‐DR expression is upregulated on hBMSCs in the presence of FGF‐2,^56^ which was also used to culture hBMSCs in this study. Grau‐Vorster et al. further reported variable and dynamic expression of HLA‐DR on hBMSCs from clinical batches cultured in human serum without additional FGF‐2 supplement.^57^ The authors have demonstrated that HLA‐DR^+^ cells maintained in vitro functional attributes such as osteogenic, adipogenic and chondrogenic differentiation potential, in unison with the data obtained in the present study, and consider HLA‐DR rather informative instead of being a criterion to define MSCs.

Overall, there are many studies with differing results in the literature, which may likely be due to differences in protocols^2^ as well as small sample sizes and different donor age groups. This highlights the need for generally accepted standards in MSC culture and related assays. In the present study, we have tried to give detailed descriptions of the methods used for isolation, culture and analysis of hBMSCs. Furthermore, the data obtained in this study are derived from a large sample size (*n* = 175), reducing variability due to individual differences of hBMSC donors.

Altogether, the present study revealed a sex bias in relation to the influence of age on hBMSC properties. The data reveal a significant decline in proliferative capacity, adipogenic differentiation potential and cell surface expression of SSEA‐4, CD146 and CD274 on hBMSCs from female, but not male, donors with increasing donor age, backed up by a large donor cohort. Therefore, donor age and sex should be carefully considered when selecting hBMSCs for the design of clinical applications as these might have a meaningful impact on the therapeutic outcome.

## CONFLICTS OF INTERESTS

The authors declare no conflicts of interests.

## AUTHOR CONTRIBUTION

**Michael Selle:** Data curation (lead); Formal analysis (lead); Investigation (equal); Methodology (equal); Software (lead); Validation (equal); Visualization (lead); Writing – original draft (lead); Writing – review & editing (equal). **jJohanna D Ehlers:** Data curation (lead); Formal analysis (lead); Investigation (equal); Methodology (equal); Validation (equal); Writing – original draft (lead); Writing – review & editing (equal). **Alina Ongsiek:** Data curation (equal); Formal analysis (equal); Investigation (equal); Writing – review & editing (equal). **Linnea Ulbrich:** Data curation (equal); Formal analysis (equal); Investigation (equal); Writing – review & editing (equal). **Weikang Ye:** Data curation (equal); Formal analysis (equal); Investigation (equal); Writing – review & editing (equal). **Zhida Jiang:** Data curation (equal); Formal analysis (equal); Investigation (equal); Writing – review & editing (equal). **Christian Krettek:** Conceptualization (supporting); Funding acquisition (supporting); Project administration (supporting); Resources (equal); Software (supporting); Supervision (equal); Writing – review & editing (equal). **Claudia Neunaber:** Conceptualization (equal); Data curation (equal); Formal analysis (equal); Funding acquisition (supporting); Investigation (equal); Methodology (equal); Project administration (lead); Resources (equal); Software (equal); Supervision (lead); Validation (equal); Visualization (lead); Writing – original draft (equal); Writing – review & editing (lead). **Sandra Noack:** Conceptualization (lead); Data curation (equal); Formal analysis (lead); Funding acquisition (lead); Investigation (lead); Methodology (lead); Project administration (lead); Resources (equal); Software (equal); Supervision (lead); Validation (lead); Visualization (equal); Writing – original draft (equal); Writing – review & editing (lead).

## Supporting information

Table S1

Table S2

Table S3

Table S4

## Data Availability

The data that support the findings of this study are available from the corresponding author upon reasonable request.
