# Supplementary material for: Influence of age on stem cells depends on the sex of the bone marrow donor
Source: J Cell Mol Med. 2022 Jan 27;26(5):1594–605. doi: 10.1111/jcmm.17201 (PMC8899192; doi:10.1111/jcmm.17201)
Supplement: Supplementary file 1 — Table S1 [file JCMM-26-1594-s001.docx]

**Supplemantary Table 1:** List of concomitant diseases of the bone marrow donors. Of the 175 donors analyzed, 134 had one or more of the diagnoses listed in the table.

| **lung/respiratory tract** | |
| --- | --- |
| COPD | 15 |
| bronchial asthma | 11 |
| sleep apnea | 4 |
| pneumonia | 1 |
| wegener's disease | 1 |
| dyspnea | 1 |
| **cardiovascular** | |
| hypertension | 55 |
| cardiac arrhythmia | 11 |
| heart failure | 10 |
| myocardial infarction | 6 |
| heart valve insufficiency | 9 |
| circulatory disorder in the heart | 4 |
| right bundle branch block | 2 |
| varicose veins | 2 |
| carotid Node | 2 |
| paroxysmal atrial fibrillation | 1 |
| aortic stenosis | 1 |
| delayed R progression | 1 |
| atrial fibrillation | 1 |
| peripheral arterial disease | 1 |
| **blood/** **coagulation** | |
| tendency to thrombosis | 6 |
| bleeding disorder | 4 |
| venous thrombosis | 3 |
| **liver** | |
| steatosis hepatis | 6 |
| **kidney / bladder** | |
| renal failure | 9 |
| shrink kidney | 1 |
| kidney cyst | 1 |
| kidney stones | 1 |
| **digestive tract** | |
| reflux | 11 |
| paralytic ileus | 1 |
| partial resection of the intestine | 3 |
| lactose intolerance | 1 |
| duodenal ulcers | 1 |
| diverticulosis | 1 |
| ulcerative colitis | 1 |
| chronic pancreatitis | 1 |
| **metabolism / diabetes** | |
| hypothyroidism | 13 |
| hyperlipoproteinemia | 13 |
| diabetes | 10 |
| hyperuricemia | 9 |
| autoimmune thyroid disease | 1 |
| gout | 1 |
| **skeleton / musculature** | |
| lumbar / cervical spine syndrome | 18 |
| arthrosis / polyarthrosis | 11 |
| osteoporosis | 5 |
| rheumatoid arthritis | 4 |
| scoliosis | 2 |
| ankylosing spondylitis | 1 |
| myositis ossificans | 1 |
| osteopenia | 1 |
| polyarthritis | 1 |
| osteomyelitis of the knee | 1 |
| **awareness / psyche** | |
| pain patient | 10 |
| depression | 10 |
| alcohol abuse | 7 |
| restless legs syndrome | 1 |
| **neurology** | |
| polyneuropathy | 6 |
| intraocular lens / cataract | 5 |
| migraine | 3 |
| epilepsy | 3 |
| glaucoma | 2 |
| hearing damage | 2 |
| spinal stenosis of the lumbar spine | 1 |
| paresis | 1 |
| parkinson's disease | 1 |
| fibromyalgia | 1 |
| multiple sclerosis | 1 |
| facial palsy | 1 |
| apoplexy | 1 |
| **allergies** | |
| antibiotics | 26 |
| pollinosis | 20 |
| band aid | 10 |
| Food | 8 |
| Nickel | 6 |
| other drugs | 6 |
| wasp /bee venom | 4 |
| animal hair, house dust | 8 |
| contrast agent / iodine | 3 |
| taxotere | 1 |
| chrome | 1 |
| chlorine | 1 |
| latex | 1 |
| **miscellaneous** | |
| tumor disease | 11 |
| sjogren's Syndrome | 1 |
| rosacea | 1 |
| kinetosis | 1 |
| desiccosis | 1 |
