# Supplementary material for: Influence of age on stem cells depends on the sex of the bone marrow donor
Source: J Cell Mol Med. 2022 Jan 27;26(5):1594–605. doi: 10.1111/jcmm.17201 (PMC8899192; doi:10.1111/jcmm.17201)
Supplement: Supplementary file 2 — Table S2 [file JCMM-26-1594-s004.docx]

**Supplementary Table 2:** Medication of the bone marrow donors. Of the 175 donors, 109 donors were taking one or more of the drugs listed in the table. The patients also received benzodiazepines, imidazolines and, if necessary, proton pump inhibitors preoperatively.

| **drug group** | |
| --- | --- |
| nonsteroidal anti-inflammatory drug | 46 |
| proton pump inhibitors | 33 |
| statine | 28 |
| diuretics | 24 |
| beta-blockers | 23 |
| opioid | 23 |
| ACE inhibitors | 21 |
| calcium channel blockers | 21 |
| angiotensin II receptor antagonists | 20 |
| thyroid preparations | 20 |
| vitamins (D, B, K), magnesium, iron | 20 |
| anticoagulants | 18 |
| non-opioid analgesics | 16 |
| benzodiazepines | 10 |
| antidepressants | 10 |
| glucocorticoid | 8 |
| alpha-Receptor Blockers | 5 |
| beta2 sympathomimetics | 5 |
| biguanide derivatives | 5 |
| anti-epileptic drugs | 4 |
| bisphosphonates | 4 |
| β₁-adrenergic blockers | 4 |
| anticonvulsants | 3 |
| COX-2 inhibitors | 3 |
| insulin | 3 |
| disease-modifying anti-inflammatory drugs | 3 |
| uricostatics | 3 |
| coumarin derivatives | 3 |
| anticholinergic | 2 |
| antiemetic | 2 |
| antiprotozoic drugs | 2 |
| azetidones | 2 |
| contraceptives | 2 |
| selective serotonin reuptake inhibitors | 2 |
| antidiabetic drugs | 1 |
| cardiac glycosides | 1 |
| potassium channel blockers | 1 |
| L-DOPA decarboxylase inhibitors | 1 |
| neuroleptics | 1 |
| non-enzymatic nitric oxide donors | 1 |
| steroid | 1 |
| synthetic anticholinergics | 1 |
| nitro-based vasodilators | 1 |
| ursodeoxycholic acid | 1 |
| serotonin agonist | 1 |
| quinine hemisulphate-2-water | 1 |
| NMDA receptor antagonists | 1 |
| laxatives | 1 |
| antibiotics | 1 |
| [vasopressin antagonist](https://flexikon.doccheck.com/de/Vasopressin-Antagonist) | 1 |
