# Supplementary material for: Influence of age on stem cells depends on the sex of the bone marrow donor
Source: J Cell Mol Med. 2022 Jan 27;26(5):1594–605. doi: 10.1111/jcmm.17201 (PMC8899192; doi:10.1111/jcmm.17201)
Supplement: Supplementary file 3 — Table S3 [file JCMM-26-1594-s003.docx]

**Supplementary Table 3: Detailed product specifications for each antibody used.**

| **Antigen** | **Conjugate** | **Isotype** | **Clone** | **Host** | **Supplier** | **Catalog** | **Antibody concentration [μg/mL]** | **Volume of antibody used per 1 x 10^5^ cells [µL]** | **Amount of antibody used [µg]** |
| --- | --- | --- | --- | --- | --- | --- | --- | --- | --- |
| CD4 | APC-Cy7 | IgG1 k | RPA-T4 | mouse | Biolegend, CA, USA | 300518 | 200 | 1.1 | 0.22 |
| CD10 | APC-Cy7 | IgG1 k | HI10a | mouse | Biolegend, CA, USA | 312212 | 400 | 0.7 | 0.28 |
| CD11b | APC | IgC1 k | ICRF44 | mouse | Biolegend, CA, USA | 301310 | 100 | 2.75 | 0.275 |
| CD11c | APC-Cy7 | IgG1 k | Bu15 | mouse | Biolegend, CA, USA | 337218 | 400 | 0.7 | 0.28 |
| CD13 | APC-Cy7 | IgG1 κ | WM15 | mouse | Biolegend, CA, USA | 301709 | 400 | 0.0275 | 0.011 |
| CD14 | APC | IgG2a κ | M5E2 | mouse | Biolegend, CA, USA | 301808 | 200 | 1.4 | 0.28 |
| CD15 | FITC | IgG1 k | W6D3 | mouse | Biolegend, CA, USA | 323004 | 400 | 0.7 | 0.28 |
| CD19 | PerCP/Cy5.5 | IgG1 k | SJ25C1 | rat | Thermo Fischer Scientific, MA, USA | 46.0198.42 | 100 | 2.75 | 0.275 |
| CD24 | PE-Cy7 | IgG2a κ | ML5 | mouse | Biolegend, CA, USA | 311120 | 200 | 1.4 | 0.28 |
| CD29 | APC | IgG1 k | TS2/16 | mouse | Biolegend, CA, USA | 303007 | 400 | 0.0275 | 0.011 |
| CD31 | FITC | IgG1 κ | WM59 | mouse | Biolegend, CA, USA | 303104 | 200 | 1.4 | 0.28 |
| CD34 | PE-Cy7 | IgG1 κ | 581 | mouse | Biolegend, CA, USA | 343516 | 100 | 2.75 | 0.275 |
| CD44 | FITC | IgG1 κ | BJ18 | mouse | Biolegend, CA, USA | 338804 | 50 | 0.22 | 0.011 |
| CD45 | APC-Cy7 | IgG1 κ | H130 | mouse | Biolegend, CA, USA | 304014 | 100 | 2.2 | 0.22 |
| CD49f | PerCP/Cy5.5 | IgG2a κ | GoH3 | rat | Biolegend, CA, USA | 313618 | 200 | 1.4 | 0.28 |
| CD56 | PE-Cy7 | IgG2a κ | MEM-188 | mouse | Biolegend, CA, USA | 304628 | 180 | 1.55 | 0.279 |
| CD73 | APC | IgG1 κ | AD2 | mouse | Biolegend, CA, USA | 344005 | 100 | 0.011 | 0.0011 |
| CD90 | PerCP/Cy5.5 | IgG1 κ | 5E 07 | mouse | Biolegend, CA, USA | 328118 | 200 | 0.55 | 0.11 |
| CD105 | PE | IgG1 κ | 43A3 | mouse | Biolegend, CA, USA | 323206 | 200 | 1.4 | 0.28 |
| CD106 | APC | IgG1 κ | STA | mouse | Biolegend, CA, USA | 303810 | 100 | 2.2 | 0.22 |
| CD117 | PerCP/Cy5.5 | IgG1 κ | 104D2 | mouse | Biolegend, CA, USA | 313214 | 200 | 1.4 | 0.28 |
| CD146 | PE-Cy7 | IgG2a κ | SHM-57 | mouse | Biolegend, CA, USA | 134714 | 50 | 5.5 | 0.275 |
| CD163 | PE | IgG1 κ | GHI/61 | mouse | Biolegend, CA, USA | 333605 | 200 | 1.4 | 0.28 |
| CD166 | FITC | IgG1 κ | 3A6 | mouse | Ancell Corporation MN, USA | 393040 | 500 | 0.44 | 0.22 |
| CD200 | PerCP/Cy5.5 | IgG1 κ | OX-104 | mouse | Biolegend, CA, USA | 329216 | 150 | 1.85 | 0.2775 |
| CD271 | PerCP/Cy5.5 | IgG1 κ | C40-1457 | mouse | Biolegend, CA, USA | 345112 | 200 | 1.4 | 0.28 |
| CD274 | PE | IgG1 κ | 29E-2A3 | mouse | Biolegend, CA, USA | 329706 | 400 | 0.7 | 0.28 |
| MSCA-1 | PE | IgG1 κ | W4A5 | mouse | Biolegend, CA, USA | 330806 | 100 | 2.75 | 0.275 |
| GD2 | PE-Cy7 | IgG2a κ | 14G2a | mouse | Biolegend, CA, USA | 357308 | 200 | 1.4 | 0.28 |
| HLA-DR | PE | IgG2b κ | LN3 | mouse | Biolegend, CA, USA | 327008 | 2 | 13.75 | 0.0275 |
| SSEA-3 | FITC | IgM κ | MC-631 | rat | Biolegend, CA, USA | 330308 | 25 | 5.5 | 0.1375 |
| SSEA-4 | FITC | IgG3 κ | MC-631 | mouse | Biolegend, CA, USA | 330410 | 160 | 11 | 1.76 |
| SSEA-5 | APC | IgG1 κ | MC-813-70 | mouse | Biolegend, CA, USA | 355208 | 400 | 0.7 | 0.28 |
| Stro-1 | APC | IgM κ | Stro-1 | mouse | Biolegend, CA, USA | 340140 | 400 | 0.6 | 0.24 |
