# Supplementary material for: Influence of age on stem cells depends on the sex of the bone marrow donor
Source: J Cell Mol Med. 2022 Jan 27;26(5):1594–605. doi: 10.1111/jcmm.17201 (PMC8899192; doi:10.1111/jcmm.17201)
Supplement: Supplementary file 4 — Table S4 [file JCMM-26-1594-s002.docx]

**Supplementary Table 4**: Further information on the surface antigens analyzed by flow cytometry.

| **Antigen** | **Function** | **Literature** |
| --- | --- | --- |
| CD4 | Through its interaction with MHC class II, CD4 is involved in thymic selection as well as the cell activation and differentiation by inducing secretion of cytokines or interacting with other surface antigens. | *Bernstein HB, Plasterer MC, Schiff SE, Kitchen CM, Kitchen S, Zack JA. CD4 expression on activated NK cells: ligation of CD4 induces cytokine expression and cell migration. J Immunol. 2006 Sep 15;177(6):3669-76. doi: 10.4049/jimmunol.177.6.3669. PMID: 16951326.* |
| CD10 | CD10 is a zinc-dependent metalloprotease, which is able to regulate inflammation or maturation of e.g. B cells. It also is involved in modulating morphogenesis of some organs. | *Maguer-Satta V, Besançon R, Bachelard-Cascales E. Concise review: neutral endopeptidase (CD10): a multifaceted environment actor in stem cells, physiological mechanisms, and cancer. Stem Cells. 2011 Mar;29(3):389-96. doi: 10.1002/stem.592. PMID: 21425402*  *Shirotani K, Tsubuki S, Iwata N, Takaki Y, Harigaya W, Maruyama K, Kiryu-Seo S, Kiyama H, Iwata H, Tomita T, Iwatsubo T, Saido TC. Neprilysin degrades both amyloid beta peptides 1-40 and 1-42 most rapidly and efficiently among thiorphan- and phosphoramidon-sensitive endopeptidases. J Biol Chem. 2001 Jun 15;276(24):21895-901. doi: 10.1074/jbc.M008511200. Epub 2001 Mar 6. PMID: 11278416.* |
| CD11b | The Tissue Stem Cell Committee of the International Society for Cellular Therapy (ISCT) define CD11b as a negative marker that MSCs typically not express. CD11b or integrin ITGAM/ITGB2 is mostly involved in inflammatory processes e.g. phagocytosis, activity of the complemental system as well as hemostasis. | *Dominici M, Le Blanc K, Mueller I, Slaper-Cortenbach I, Marini F, Krause D, Deans R, Keating A, Prockop Dj, Horwitz E. Minimal criteria for defining multipotent mesenchymal stromal cells. The International Society for Cellular Therapy position statement. Cytotherapy. 2006;8(4):315-7. doi: 10.1080/14653240600855905. PMID: 16923606.*  *Losse J, Zipfel PF, Józsi M. Factor H and factor H-related protein 1 bind to human neutrophils via complement receptor 3, mediate attachment to Candida albicans, and enhance neutrophil antimicrobial activity. J Immunol. 2010 Jan 15;184(2):912-21. doi: 10.4049/jimmunol.0901702. Epub 2009 Dec 11. PMID: 20008295.*  *Bai M, Grieshaber-Bouyer R, Wang J, Schmider AB, Wilson ZS, Zeng L, Halyabar O, Godin MD, Nguyen HN, Levescot A, Cunin P, Lefort CT, Soberman RJ, Nigrovic PA. CD177 modulates human neutrophil migration through activation-mediated integrin and chemoreceptor regulation. Blood. 2017 Nov 9;130(19):2092-2100. doi: 10.1182/blood-2017-03-768507. Epub 2017 Aug 14. PMID: 28807980; PMCID: PMC5680608.* |
| CD11c | CD11c, also known as Integrin, alpha X (complement component 3 receptor 4 subunit) (ITGAX) is typically expressed on cells regarding to the immune system for example phagocytes. Its expression was also detected on chondrocytes, where it plays a role in ossification. | *Kiernan CH, KleinJan A, Peeters M, Wolvius EB, Farrell E, Brama PAJ. Allogeneic chondrogenically differentiated human bone marrow stromal cells do not induce dendritic cell maturation. J Tissue Eng Regen Med. 2018 Jun;12(6):1530-1540. doi: 10.1002/term.2682. PMID: 29702747; PMCID: PMC6032931.* |
| CD13 | Alanin-Aminopeptidase is expressed on mesenchymal stem cells. It also represents one of the earliest markers of the myeloid cell series and seems to be involved in regulatory processes regarding angiogenesis. | *Cunningham MD, Shapiro RA, Seachord C, Ratcliffe K, Cassiano L, Darveau RP. CD14 employs hydrophilic regions to "capture" lipopolysaccharides. J Immunol. 2000 Mar 15;164(6):3255-63. doi: 10.4049/jimmunol.164.6.3255. PMID: 10706718.*  *Manukyan M, Triantafilou K, Triantafilou M, Mackie A, Nilsen N, Espevik T, Wiesmüller KH, Ulmer AJ, Heine H. Binding of lipopeptide to CD14 induces physical proximity of CD14, TLR2 and TLR1. Eur J Immunol. 2005 Mar;35(3):911-21. doi: 10.1002/eji.200425336. PMID: 15714590.* |
| CD14 | The ISCT define CD14 as a negative marker that MSCs typically not express. A surface antigen which is mostly excerpted by immuno potent cells. It can be used to screen for contamination within the hBMSCs. | *Dominici M, Le Blanc K, Mueller I, Slaper-Cortenbach I, Marini F, Krause D, Deans R, Keating A, Prockop Dj, Horwitz E. Minimal criteria for defining multipotent mesenchymal stromal cells. The International Society for Cellular Therapy position statement. Cytotherapy. 2006;8(4):315-7. doi: 10.1080/14653240600855905. PMID: 16923606.*  *Pilz GA, Braun J, Ulrich C, Felka T, Warstat K, Ruh M, Schewe B, Abele H, Larbi A, Aicher WK. Human mesenchymal stromal cells express CD14 cross-reactive epitopes. Cytometry A. 2011 Aug;79(8):635-45. doi: 10.1002/cyto.a.21073. Epub 2011 Jul 6. PMID: 21735544.*  *Satoh T, Knowles A, Li MS, Sun L, Tooze JA, Zabucchi G, Spry CJ. Expression of lacto-N-fucopentaose III (CD15)- and sialyl-Lewis X-bearing molecules and their functional properties in eosinophils from patients with the idiopathic hypereosinophilic syndrome. Immunology. 1994 Oct;83(2):313-8. PMID: 7835953; PMCID: PMC1414932.* |
| CD15 | CD15, also known as stage specific antigen 1 (SSEA1), is involved in cellular interactions during the immune response. Typically, it is not expressed on mesenchymal stem cells. | *Atz ME, Rollins B, Vawter MP. NCAM1 association study of bipolar disorder and schizophrenia: polymorphisms and alternatively spliced isoforms lead to similarities and differences. Psychiatr Genet. 2007 Apr;17(2):55-67. doi: 10.1097/YPG.0b013e328012d850. PMID: 17413444; PMCID: PMC2077086.* |
| CD19 | The ISCT define CD19 as a negative marker that MSCs typically not express. CD19 is expressed on B-lymphocytes and affects the signaling threshold of receptors that regulate B lymphocyte selection, activation, and differentiation. | *Dominici M, Le Blanc K, Mueller I, Slaper-Cortenbach I, Marini F, Krause D, Deans R, Keating A, Prockop Dj, Horwitz E. Minimal criteria for defining multipotent mesenchymal stromal cells. The International Society for Cellular Therapy position statement. Cytotherapy. 2006;8(4):315-7. doi: 10.1080/14653240600855905. PMID: 16923606.*  *Engel P, Zhou LJ, Ord DC, Sato S, Koller B, Tedder TF. Abnormal B lymphocyte development, activation, and differentiation in mice that lack or overexpress the CD19 signal transduction molecule. Immunity. 1995 Jul;3(1):39-50. doi: 10.1016/1074-7613(95)90157-4. PMID: 7542548.* |
| CD24 | Is among others expressed on B-lymphocytes where it modulates the B-cell-receptor activity. | *Hough MR, Rosten PM, Sexton TL, Kay R, Humphries RK. Mapping of CD24 and homologous sequences to multiple chromosomal loci. Genomics. 1994 Jul 1;22(1):154-61. doi: 10.1006/geno.1994.1356. PMID: 7959762.* |
| CD29 | CD29 is linked to CD49d and is expressed on mesenchymal cells. It plays a role in recruiting and migration as well as differentiation of mesenchymal cells. | *Ode A, Schoon J, Kurtz A, Gaetjen M, Ode JE, Geissler S, Duda GN. CD73/5'-ecto-nucleotidase acts as a regulatory factor in osteo-/chondrogenic differentiation of mechanically stimulated mesenchymal stromal cells. Eur Cell Mater. 2013 Jan 8;25:37-47. doi: 10.22203/ecm.v025a03. PMID: 23300031.* |
| CD31 | CD31 is assigned to the superfamily of immunoglobulins. Typically it is not expressed on mesenchymal stem cells. | *Rege TA, Hagood JS. Thy-1 as a regulator of cell-cell and cell-matrix interactions in axon regeneration, apoptosis, adhesion, migration, cancer, and fibrosis. FASEB J. 2006 Jun;20(8):1045-54. doi: 10.1096/fj.05-5460rev. PMID: 16770003.* |
| CD34 | The ISCT define CD34 as a negative marker that MSCs typically not express.  CD34 is a typically marker for hematopoietic cells, but was detected as a surface antigen on MSCs, too. In addition, CD34 was found in several cancer entities. CD34+ cells can have a positive effect on different deceases. | *Dominici M, Le Blanc K, Mueller I, Slaper-Cortenbach I, Marini F, Krause D, Deans R, Keating A, Prockop Dj, Horwitz E. Minimal criteria for defining multipotent mesenchymal stromal cells. The International Society for Cellular Therapy position statement. Cytotherapy. 2006;8(4):315-7. doi:*  *10.1080/14653240600855905. PMID: 16923606.*  Sidney LE, Branch MJ, Dunphy SE, Dua HS, Hopkinson A. Concise review: evidence for CD34 as a common marker for diverse progenitors. Stem Cells. 2014 Jun;32(6):1380-9. doi: 10.1002/stem.1661. PMID: 24497003; PMCID: PMC4260088.  Srivastava A, Bapat M, Ranade S, Srinivasan V, Murugan P, Manjunath S, Thamaraikannan P, Abraham S. Multiple injections of in vitro expanded autologous bone marrow stem cells for cervical level spinal cord injury - a case report. J Stem Cells Regen Med. 2010 Oct 30;6(3):175-6. PMID: 24693165. |
| CD44 | CD44 is also called phagocytotic glykoprotein 1 (Pgp-1) and interacts with parts of the extracellular matrix e.g. hyaluronic acid. It is regularly expressed on mesenchymal stem cells | *Nakamura H, Yukita A, Ninomiya T, Hosoya A, Hiraga T, Ozawa H. Localization of Thy-1-positive cells in the perichondrium during endochondral ossification. J Histochem Cytochem. 2010 May;58(5):455-62. doi: 10.1369/jhc.2010.955393. Epub 2010 Feb 1. PMID: 20124093; PMCID: PMC2857817.* |
| CD45 | The ISCT define CD45 as a negative marker that MSCs typically not express. CD45 is expressed by hematopoetic cells and is not related to hMSCs. | *Dominici M, Le Blanc K, Mueller I, Slaper-Cortenbach I, Marini F, Krause D, Deans R, Keating A, Prockop Dj, Horwitz E. Minimal criteria for defining multipotent mesenchymal stromal cells. The International Society for Cellular Therapy position statement. Cytotherapy. 2006;8(4):315-7. doi:*  *10.1080/14653240600855905. PMID: 16923606.*  *Cybulsky MI, Fries JW, Williams AJ, Sultan P, Davis VM, Gimbrone MA Jr, Collins T. Alternative splicing of human VCAM-1 in activated vascular endothelium. Am J Pathol. 1991 Apr;138(4):815-20. PMID: 1707234; PMCID: PMC1886101.* |
| CD49f | CD49f is part of a heterodimer and is expressed on thrombocytes and epithelial cells. Via its participation in the structure of hemidesmosomes, mutations in its structure plays a role in developing epidermolysis bullosa. | *Sá R, Miranda C, Carvalho F, Barros A, Sousa M. Expression of stem cell markers: OCT4, KIT, ITGA6, and ITGB1 in the male germinal epithelium. Syst Biol Reprod Med. 2013 Oct;59(5):233-43. doi: 10.3109/19396368.2013.804964. Epub 2013 Jun 13. PMID: 23758503.*  *Tachibana I, Bodorova J, Berditchevski F, Zutter MM, Hemler ME. NAG-2, anovel transmembrane-4 superfamily (TM4SF) protein that complexes with integrinsand other TM4SF proteins. J Biol Chem. 1997 Nov 14;272(46):29181-9. doi:10.1074/jbc.272.46.29181. PMID: 9360996.* |
| CD56 | CD56 is typically expressed by MSCs. Its expression can also be detected on natural killer cells. | *Solchaga LA, Penick K, Goldberg VM, Caplan AI, Welter JF. Fibroblast growth factor-2 enhances proliferation and delays loss of chondrogenic potential in human adult bone-marrow-derived mesenchymal stem cells. Tissue Eng Part A. 2010 Mar;16(3):1009-19. doi: 10.1089/ten.TEA.2009.0100. PMID: 19842915; PMCID: PMC2862658.* |
| CD73 | The Tissue Stem Cell Committee of the International Society for Cellular Therapy (ISCT) defines CD73 as a positive marker that MSCs typically has to express. CD73 is expressed among others on MSCs and is able to generate nucleosides through dephosphorization. | *Dominici M, Le Blanc K, Mueller I, Slaper-Cortenbach I, Marini F, Krause D, Deans R, Keating A, Prockop Dj, Horwitz E. Minimal criteria for defining multipotent mesenchymal stromal cells. The International Society for Cellular Therapy position statement. Cytotherapy. 2006;8(4):315-7. doi: 10.1080/14653240600855905. PMID: 16923606.*  *Kebir A, Harhouri K, Guillet B, Liu JW, Foucault-Bertaud A, Lamy E, Kaspi E, Elganfoud N, Vely F, Sabatier F, Sampol J, Pisano P, Kruithof EK, Bardin N, Dignat-George F, Blot-Chabaud M. CD146 short isoform increases the proangiogenic potential of endothelial progenitor cells in vitro and in vivo. Circ Res. 2010*  *Jul 9;107(1):66-75. doi: 10.1161/CIRCRESAHA.109.213827. Epub 2010 May 6. PMID: 20448216.* |
| CD90 | The ISCT define CD90 as a positive marker that MSCs typically has to express. CD90 is typically expressed on MSCs where it is part of the chondrogenic ossification. | *Dominici M, Le Blanc K, Mueller I, Slaper-Cortenbach I, Marini F, Krause D, Deans R, Keating A, Prockop Dj, Horwitz E. Minimal criteria for defining multipotent mesenchymal stromal cells. The International Society for Cellular Therapy position statement. Cytotherapy. 2006;8(4):315-7. doi:10.1080/14653240600855905. PMID: 16923606.*  *Halfon S, Abramov N, Grinblat B, Ginis I. Markers distinguishing mesenchymal stem cells from fibroblasts are downregulated with passaging. Stem Cells Dev. 2011 Jan;20(1):53-66. doi: 10.1089/scd.2010.0040. Epub 2010 Oct 26. PMID: 20528146.* |
| CD105 | The ISCT define CD105 as a positive marker that MSCs typically has to express. Compared to pluripotent stem cells, CD105 is increasingly expressed on the cell surface of MSCs with progressed mesenchymal differentiation. | *Dominici M, Le Blanc K, Mueller I, Slaper-Cortenbach I, Marini F, Krause D, Deans R, Keating A, Prockop Dj, Horwitz E. Minimal criteria for defining multipotent mesenchymal stromal cells. The International Society for Cellular Therapy position statement. Cytotherapy. 2006;8(4):315-7. doi: 10.1080/14653240600855905. PMID: 16923606.*  *Lee MK, Pardoux C, Hall MC, Lee PS, Warburton D, Qing J, Smith SM, Derynck R. TGF-beta activates Erk MAP kinase signalling through direct phosphorylation of ShcA. EMBO J. 2007 Sep 5;26(17):3957-67. doi: 10.1038/sj.emboj.7601818. Epub 2007 Aug 2. PMID: 17673906; PMCID: PMC1994119.* |
| CD106 | CD106 also known as VCAM-1 (Vascular cell adhesion molecule 1) is expressed by some mesenchymal cells. Its origin function is known as regulating diapedesis of white blood cells through endothelia while inflammation. | Yang ZX, Han ZB, Ji YR, Wang YW, Liang L, Chi Y, Yang SG, Li LN, Luo WF, Li JP, Chen DD, Du WJ, Cao XC, Zhuo GS, Wang T, Han ZC. CD106 identifies a subpopulation of mesenchymal stem cells with unique immunomodulatory properties. PLoS One. 2013;8(3):e59354. doi: 10.1371/journal.pone.0059354. Epub 2013 Mar 12.PMID: 23555021; PMCID: PMC3595282. |
| CD117 | The cytokine receptor c-Kit or CD117 is expressed on the surface of hematopoietic stem cells or other cell types and can be involved with some types of cancer. | Edling CE, Hallberg B. c-Kit--a hematopoietic cell essential receptor tyrosine kinase. Int J Biochem Cell Biol. 2007;39(11):1995-8. doi: 10.1016/j.biocel.2006.12.005. Epub 2007 Jan 20. PMID: 17350321. |
| CD146 | The expression of CD146, also known as MCAM (melanoma-associated cell adhesion molecule), on MSCs has been associated with their differentiation potential. | Russell KC, Phinney DG, Lacey MR, Barrilleaux BL, Meyertholen KE, O'Connor KC. In vitro high-capacity assay to quantify the clonal heterogeneity in trilineage potential of mesenchymal stem cells reveals a complex hierarchy of lineage commitment. Stem Cells. 2010 Apr;28(4):788-98. doi: 10.1002/stem.312. PMID: 20127798. |
| CD163 | CD163 is a scavenger receptor that has a high affinity for the hemoglobin-haptoglobin complex. It is also defined as a marker for monocytes and macrophages and functions as an innate immune sensor for Gram-positive and Gram-negative bacteria. | Kristiansen M, Graversen JH, Jacobsen C, Sonne O, Hoffman HJ, Law SK, Moestrup SK. Identification of the haemoglobin scavenger receptor. Nature. 2001 Jan 11;409(6817):198-201. doi: 10.1038/35051594. PMID: 11196644.  *Lau SK, Chu PG, Weiss LM. CD163: a specific marker of macrophages in paraffin-embedded tissue samples. Am J Clin Pathol. 2004 Nov;122(5):794-801. doi: 10.1309/QHD6-YFN8-1KQX-UUH6. PMID: 15491976.*  Fabriek BO, van Bruggen R, Deng DM, Ligtenberg AJ, Nazmi K, Schornagel K, Vloet RP, Dijkstra CD, van den Berg TK. The macrophage scavenger receptor CD163 functions as an innate immune sensor for bacteria. Blood. 2009 Jan 22;113(4):887-92. doi: 10.1182/blood-2008-07-167064. Epub 2008 Oct 10. PMID: 18849484.  Van Gorp H, Delputte PL, Nauwynck HJ. Scavenger receptor CD163, a Jack-of- all-trades and potential target for cell-directed therapy. Mol Immunol. 2010 Apr;47(7-8):1650-60. doi: 10.1016/j.molimm.2010.02.008. Epub 2010 Mar 17. PMID: 20299103. |
| CD166 | CD166, also known as ALCAM (activated leukocyte cell adhesion molecule), is typically expressed on MSCs and is important for the cell to cell adhesion of homophil and heterophil Interaction (with CD6). | *Tang C, Lee AS, Volkmer JP, Sahoo D, Nag D, Mosley AR, Inlay MA, Ardehali R, Chavez SL, Pera RR, Behr B, Wu JC, Weissman IL, Drukker M. An antibody against SSEA-5 glycan on human pluripotent stem cells enables removal of teratoma- forming cells. Nat Biotechnol. 2011 Aug 14;29(9):829-34. doi: 10.1038/nbt.1947. PMID: 21841799; PMCID: PMC3537836.* |
| CD200 | As a ligand for an immune inhibitory receptor, CD200 is expressed on myeloid and lymphoid cells and is defined as an important immunological checkpoint. Furthermore, it is considered as independent factor for multiple myeloma and acute myeloid leukemia and predicts reduced overall survival when expressed. | *Rijkers ES, de Ruiter T, Baridi A, Veninga H, Hoek RM, Meyaard L. The inhibitory CD200R is differentially expressed on human and mouse T and B lymphocytes. Mol Immunol. 2008 Feb;45(4):1126-35. doi: 10.1016/j.molimm.2007.07.013. Epub 2007 Aug 21. PMID: 17714785.*  *Moreaux J, Hose D, Reme T, Jourdan E, Hundemer M, Legouffe E, Moine P, Bourin P, Moos M, Corre J, Möhler T, De Vos J, Rossi JF, Goldschmidt H, Klein B. CD200 is a new prognostic factor in multiple myeloma. Blood. 2006 Dec 15;108(13):4194-7. doi: 10.1182/blood-2006-06-029355. Epub 2006 Aug 31. Erratum in: Blood. 2007 Apr 1;109(7):2717. PMID: 16946299.* |
| CD271 | CD271, also known as low-affinity nerve growth factor receptor, was mentioned as specific marker for multipotent hBMSCs. It is markable, that its detection is dependent on its source, e.g. umbilical cord blood aspiration. | *Quirici N, Soligo D, Bossolasco P, Servida F, Lumini C, Deliliers GL. Isolation of bone marrow mesenchymal stem cells by anti-nerve growth factor receptor antibodies. Exp Hematol. 2002 Jul;30(7):783-91. doi: 10.1016/s0301-472x(02)00812-3. PMID: 12135677.* |
| CD274 | CD274, also known as Programmed Cell Death 1 Ligand 1 (PDL1), is expressed by some mesenchymal cells and plays a role in anticipate prognosis and initiating immunotherapy regarding different types of cancer. | *David Kerr, Rebecca Johnso: Immunotherapy for Gastrointestinal Cancer. Springer, 2017,* [*ISBN 978-3-319-43063-8*](https://de.wikipedia.org/wiki/Spezial:ISBN-Suche/9783319430638)*, S. 129. (Book)* |
| MSCA-1 | Mesenchymal stromal cell antigen-1 (MSCA-1) is identical to tissue nonspecific alkaline phosphatase (TNAP), an ectoenzyme expressed in liver, bone, kidney, and embryonic stem (ES) cells. TNAP is involved in a number of processes related to MSC features, such as cell differentiation, immunomodulatory properties or disease occurrence. | *Sobiesiak M, Sivasubramaniyan K, Hermann C, Tan C, Orgel M, Treml S, Cerabona F, de Zwart P, Ochs U, Müller CA, Gargett CE, Kalbacher H, Bühring HJ. The mesenchymal stem cell antigen MSCA-1 is identical to tissue non-specific alkaline phosphatase. Stem Cells Dev. 2010 May;19(5):669-77. doi: 10.1089/scd.2009.0290. PMID: 19860546.*  *Estève D, Galitzky J, Bouloumié A, Fonta C, Buchet R, Magne D. Multiple Functions of MSCA-1/TNAP in Adult Mesenchymal Progenitor/Stromal Cells. Stem Cells Int. 2016;2016:1815982. doi: 10.1155/2016/1815982. Epub 2015 Dec 29. PMID: 26839555; PMCID: PMC4709781.* |
| GD2 | GD2 is a disialoganglioside that is expressed in a tumor specific manner. In particular, it is expressed in tumors of neuroectodermal origin, including human neuroblastomas and melanomas. It is less commonly found in normal tissues, particularly in the cerebellum and peripheral nerves of humans. | Wierzbicki A, Gil M, Ciesielski M, Fenstermaker RA, Kaneko Y, Rokita H, Lau JT, Kozbor D. Immunization with a mimotope of GD2 ganglioside induces CD8+ T cells that recognize cell adhesion molecules on tumor cells. J Immunol. 2008 Nov 1;181(9):6644-53. doi: 10.4049/jimmunol.181.9.6644. PMID: 18941255; PMCID: PMC2730120. |
| HLA-DR | The ISCT define HLA-DR as a negative marker that MSCs typically not express. HLA-DR triggers a T-(helper)-cell response by presenting potentially foreign peptide antigens to the immune system and inducing antibody formation. HLA-DR is typically found in antigen-presenting cells (macrophages, B cells, and dendritic cells) and is therefore considered as a marker of immune stimulation. | *Dominici M, Le Blanc K, Mueller I, Slaper-Cortenbach I, Marini F, Krause D, Deans R, Keating A, Prockop Dj, Horwitz E. Minimal criteria for defining multipotent mesenchymal stromal cells. The International Society for Cellular Therapy position statement. Cytotherapy. 2006;8(4):315-7. doi: 10.1080/14653240600855905. PMID: 16923606.*  *Marsh SG, Albert ED, Bodmer WF, Bontrop RE, Dupont B, Erlich HA, Fernández- Viña M, Geraghty DE, Holdsworth R, Hurley CK, Lau M, Lee KW, Mach B, Maiers M, Mayr WR, Müller CR, Parham P, Petersdorf EW, Sasazuki T, Strominger JL, Svejgaard A, Terasaki PI, Tiercy JM, Trowsdale J. Nomenclature for factors of the HLA system, 2010. Tissue Antigens. 2010 Apr;75(4):291-455. doi: 10.1111/j.1399-0039.2010.01466.x. PMID: 20356336; PMCID: PMC2848993.* |
| SSEA-3 | Stage-specific embryonic antigen 3 (SSEA-3) is a glycosphingolipid. Sphingolipids in general represent a key role in cell signaling. | Futerman AH, Hannun YA. The complex life of simple sphingolipids. EMBO Rep. 2004 Aug;5(8):777-82. doi: 10.1038/sj.embor.7400208. PMID: 15289826; PMCID: PMC1299119. |
| SSEA-4 | Stage-specific embryonic antigen (SSEA-4) is typically expressed on hBMSCs and can help to identify adult MSCs. It also gives hint for undifferentiated pluripotent human embryonic stem cells. | *Gang EJ, Bosnakovski D, Figueiredo CA, Visser JW, Perlingeiro RC. SSEA-4 identifies mesenchymal stem cells from bone marrow. Blood. 2007 Feb 15;109(4):1743-51. doi: 10.1182/blood-2005-11-010504. Epub 2006 Oct 24. PMID: 17062733.* |
| SSEA-5 | Stage-specific embryonic antigen (SSEA)-5 is a pluripotency surface marker expressed within the blastocyst and on human pluripotent stem cells. During differentiation, SSEA-5 expressions continuously decreases and is therefore used as a marker for undifferentiated pluripotent stem cells. | *Tang C, Lee AS, Volkmer JP, Sahoo D, Nag D, Mosley AR, Inlay MA, Ardehali R, Chavez SL, Pera RR, Behr B, Wu JC, Weissman IL, Drukker M. An antibody against SSEA-5 glycan on human pluripotent stem cells enables removal of teratoma- forming cells. Nat Biotechnol. 2011 Aug 14;29(9):829-34. doi: 10.1038/nbt.1947. PMID: 21841799; PMCID: PMC3537836.* |
| Stro-1 | Stro-1 is considered a specific marker for hBMSCs. Its function is not clear in detail. It was one of the first detected surface antigens identifying hBMSCs. | *Watt SM, Karhi K, Gatter K, Furley AJ, Katz FE, Healy LE, Altass LJ, Bradley NJ, Sutherland DR, Levinsky R, et al. Distribution and epitope analysis of the cell membrane glycoprotein (HPCA-1) associated with human hemopoietic progenitor cells. Leukemia. 1987 May;1(5):417-26. PMID: 2444831.* |
